# Global processing in amblyopia: a review

**DOI:** 10.3389/fpsyg.2014.00583

**Published:** 2014-06-17

**Authors:** Lisa M. Hamm, Joanna Black, Shuan Dai, Benjamin Thompson

**Affiliations:** ^1^Department of Optometry and Vision Science, University of AucklandAuckland, New Zealand; ^2^Department of Ophthalmology, Starship Children’s HospitalAuckland, New Zealand; ^3^Department of Ophthalmology, University of AucklandAuckland, New Zealand; ^4^Department of Optometry and Vision Science, University of WaterlooWaterloo, Canada

**Keywords:** amblyopia, visual deprivation, psychophysics, global processing, motion perception, form perception

## Abstract

Amblyopia is a neurodevelopmental disorder of the visual system that is associated with disrupted binocular vision during early childhood. There is evidence that the effects of amblyopia extend beyond the primary visual cortex to regions of the dorsal and ventral extra-striate visual cortex involved in visual integration. Here, we review the current literature on global processing deficits in observers with either strabismic, anisometropic, or deprivation amblyopia. A range of global processing tasks have been used to investigate the extent of the cortical deficit in amblyopia including: global motion perception, global form perception, face perception, and biological motion. These tasks appear to be differentially affected by amblyopia. In general, observers with unilateral amblyopia appear to show deficits for local spatial processing and global tasks that require the segregation of signal from noise. In bilateral cases, the global processing deficits are exaggerated, and appear to extend to specialized perceptual systems such as those involved in face processing.

## INTRODUCTION

Amblyopia is a neurodevelopmental disorder of the visual system. It is caused by abnormal visual experience during early childhood, and it results in persistent deficits in cortical processing even when normal input to the visual cortex is restored (see Wong (2012) and Birch (2013) for recent reviews). Amblyopia is typically divided into three categories based on the eye disorder responsible for disrupting visual development. The most common amblyogenic factors are strabismus or “squint” (misalignment of the visual axes causing decorrelated input from the two eyes to the visual cortex), anisometropia (unequal refractive error causing a blurred retinal image) and deprivation (a physical obstruction, such as a cataract or other media opacity preventing patterned visual input). Amblyopia associated with the presence of multiple amblyogenic factors is known as mixed mechanism amblyopia, the most common combination being strabismus and anisometropia. For review see Simons (2005), Holmes and Clarke (2006), and Wu and Hunter (2006).

Amblyopia associated with strabismus and/or refractive error is typically unilateral with acuity loss in the amblyopic, but not the non-amblyopic or “fellow” eye. In addition to acuity loss, there are significant disruptions to binocular vision. For example, stereoscopic depth perception is often impaired or absent (Agrawal et al., 2006; Wallace et al., 2011b), binocular summation is disrupted (Thompson et al., 2011; Pineles et al., 2013) and suppression of inputs from the amblyopic eye may occur (Sireteanu and Fronius, 1981; Sireteanu, 1982; Sengpiel et al., 2006; Mansouri et al., 2008). Deprivation amblyopia can be unilateral or bilateral, with acuity losses in one or both eyes despite resolution of ocular pathology. Less is known about the degree to which binocular function is affected in deprivation amblyopia, although it is clear that stereopsis is often compromised (for example, Tytla et al., 1993).

Amblyopia is of interest both from a clinical and a neuroscientific perspective (Thompson, 2012). Clinically, amblyopia caused by strabismus, anisometropia, or both affects approximately 3% of the population (Brown et al., 2000). Additionally, potentially amblyogenic cataracts have an estimated incidence of 3–4.5 per 10,000 births (Holmes et al., 2003) which is likely to be higher in low-income countries (Courtright, 2012). Unilateral amblyopia can be treated effectively in early childhood, when the visual cortex is still developing, by providing best refractive correction (Stewart et al., 2004; Cotter et al., 2012) followed by occlusion of the fellow eye (Wallace, 2006; Wallace et al., 2011a). Although this treatment improves amblyopic eye visual acuity, compliance can be problematic (Smith et al., 1995; Loudon et al., 2006; Tjiam et al., 2011), long treatment periods are often required (Awan et al., 2010) and improvements in binocular visual function are limited (Wallace et al., 2011b). In addition, standard treatment has traditionally been thought to be ineffective in older children and adults due to insufficient visual cortex plasticity, meaning that older patients with amblyopia are often left untreated. This traditional view, however, does not account for evidence that vision can be recovered in at least a subset of adults with amblyopia (Birnbaum et al., 1977; Vereecken and Brabant, 1984; Simmers and Gray, 1999; El Mallah et al., 2000; Rahi et al., 2002). Little is known about the treatment of deprivation amblyopia (for example, Hatt et al., 2009) although similar strategies are often employed.

From a neuroscientific perspective, experimentally induced amblyopia is one of the most widely used animal models for investigating the mechanisms underlying visual cortex development and plasticity. This is highlighted by the work of Nobel laureates Wiesel and Hubel (1963, 1965), Hubel and Wiesel (1965). Animal models of amblyopia allow for the role of sensory experience in cortical development to be explored, and enable investigations into the ability of environmental and pharmacological manipulations to induce neuroplasticity in the adult brain (Vetencourt et al., 2008; Montey and Quinlan, 2011; O’Leary et al., 2012; van Versendaal et al., 2012; Duffy and Mitchell, 2013). Given the central role of neuroplasticity in recovery from a wide range of neurological and psychiatric disorders, studies using amblyopia as a model have the potential to generate new intervention strategies that are applicable to a wide range of disorders (for example, Cramer et al., 2011; Maurer and Hensch, 2012).

Amblyopia is also relevant to cognitive neuroscience as human amblyopia provides important insights into the role of coordinated, binocular visual experience in visual development. Indeed, psychophysical studies of amblyopia have revealed a broad range of visual deficits associated with amblyopia that are not limited to impaired visual acuity and binocular function. These can loosely be grouped into impaired perception of individual elements within the visual scene (“local processing”) and deficits affecting the integration of multiple elements across space and time (“global processing”; for a brief overview see Dakin, 2009). Impaired local processing is often linked to abnormalities within the primary visual cortex (V1) which contains cells that tend to have relatively small receptive fields and therefore sample limited regions of the retinal image. Global processing impairments, on the other hand, are thought to involve extra-striate areas (cortical areas beyond V1). These areas tend to have larger receptive fields, integrate signals emanating from earlier stages of the visual pathway, and play a role in signal/noise segregation (for example, Born and Bradley, 2005).

Global processing has long been conceptualized in light of the parallel processing hypothesis (Haxby et al., 1991; Goodale and Milner, 1992; Van Essen and Gallant, 1994). This hypothesis proposes that dorsal extra-striate visual areas, such as motion-sensitive area V5/MT, are specialized for representing the location and movement of objects and therefore provide a foundation for visuomotor coordination. This processing pathway is referred to as the “dorsal” or “vision for action” stream and extends from the occipital to the parietal lobe. The second pathway, known as the “ventral” or “vision for recognition” stream, includes ventral regions of the occipital and temporal lobes and is thought to be specialized for the processing of form which supports object recognition. The ventral stream is interconnected with areas in the temporal lobe which deal with non-visual functions, such as language and memory. The parallel processing hypothesis has provided a useful framework for the investigation of global processing in visual development (for example, Braddick et al., 2003; Parrish et al., 2005) and amblyopia (for example, Simmers et al., 2006; Husk and Hess, 2013) although the extent of cross-talk between the two streams is yet to be fully understood (Braddick et al., 2000; Schenk and McIntosh, 2010; de Haan and Cowey, 2011; Gilaie-Dotan et al., 2013).

While it is now well accepted that amblyopia results in global processing deficits, a key question remains: are global processing deficits simply an extension of local processing deficits or is global processing impaired in its own right? Answering this question will provide important insights into the role of sensory experience in cortical development, plasticity, and the visual deficits experienced by patients with amblyopia. Psychophysical studies of amblyopia in humans have addressed this issue and are the focus of this review, which aims to (1) summarize the psychophysical studies investigating local and global processing in amblyopia, (2) assess whether global processing deficits vary across the different sub-types of amblyopia, (psychophysical studies tend to focus on unilateral strabismic and anisometropic amblyopia, or visual deprivation due to childhood cataract) and (3) consider the implications of global processing deficits for the treatment of amblyopia. A summary figure is provided at the end of the review which is designed to identify key themes.

## LOCAL PROCESSING DEFICITS IN AMBLYOPIA

### LOCAL SPATIAL PROCESSING

**FIGURE 1:**
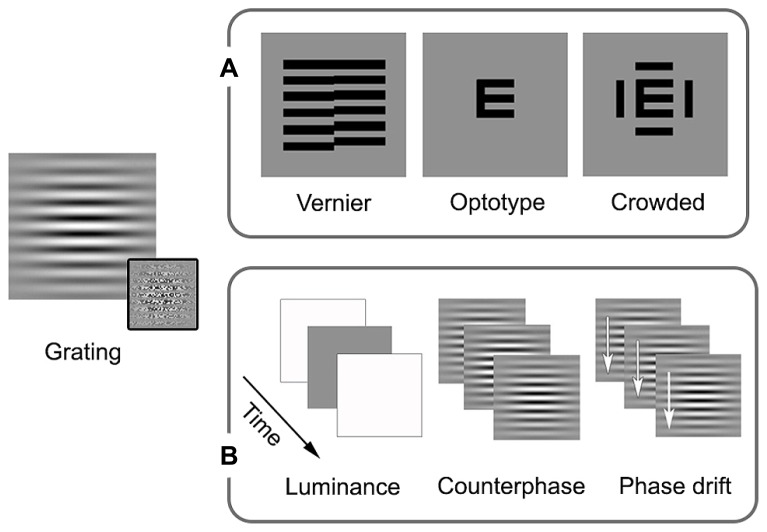
**Schematic examples of stimuli commonly used in studies of local processing and visual acuity in amblyopia.** Sinusoidal gratings convolved with Gaussian spatial window (Gabor patches) allow for the presentation of specific spatial frequencies (spatially narrowband). First-order stimuli are defined by luminance, while second-order stimuli are defined by something other than luminance, the example in the inset is of variation in contrast. These stimuli can be modulated spatially or temporally. **(A)** In addition to orientation, spatial processing measures can include vernier, optotype, and crowded optotype acuities. Broadband examples are shown as these stimuli are most often used in clinical settings (narrowband stimuli can also be used). **(B)** Examples of temporal modulation are luminance alterations (changing luminance over time, giving the impression of a flicker), counterphase modulation (exchanging peak and trough luminance over time, giving the impression of a flicker or a jump in spacing), and drift (shifting the phase over time, allowing for the perception of local motion). See text for more details.

Amblyopia is primarily thought of as a disorder of spatial vision, as reflected by the clinical emphasis on reduced visual acuity. Psychophysical studies have shown that amblyopia affects multiple aspects of spatial vision including contrast sensitivity, hyperacuity (for example, vernier acuity), crowding, and second-order spatial processing (see **Figure 1A** for schematic examples of the stimuli commonly used in psychophysical and clinical studies of spatial vision in amblyopia).

#### First-order spatial processing

Early psychophysical studies reported reduced contrast sensitivity for mid and high spatial frequencies in the amblyopic eyes of observers with strabismic (Hess and Howell, 1977; Hess et al., 1978a; Levi and Harwerth, 1978) and anisometropic (Freeman and Thibos, 1975; Levi and Harwerth, 1978) amblyopia. Deprivation amblyopia has also been found to impair contrast sensitivity and these impairments can be severe (Hess and Howell, 1977; Levi and Harwerth, 1980; Hess et al., 1981). Interestingly, the contrast sensitivity and acuity deficits in eyes with deprivation amblyopia tend to be more pronounced in unilateral then bilateral cases (Birch et al., 1998; Ellemberg et al., 1999, 2000). However, the extent of the vision loss is strongly tied to the age of onset and duration of deprivation (Birch et al., 1993; Birch and Stager, 1996).

#### Hyperacuity and crowding

**FIGURE 2:**
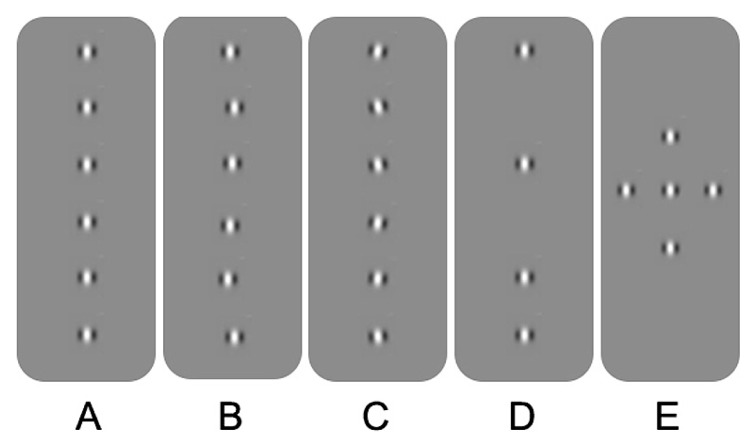
**Schematic overview of the different explanations for positional uncertainty in amblyopia. (A)** Gabor patches aligned in position and orientation. **(B)** Spatial disarray due to jitter in topographical position. **(C)** Spatial disarray due to jitter in orientation. **(D)** Undersampling of the Gabor array. **(E)** A Gabor array that would engage multiple lateral interactions between adjacent neurons. See text for more details.

Spatial processing deficits in observers with amblyopia have also been found for tasks involving hyperacuity and crowding. Hyperacuity refers to the ability to detect spatial details that are beyond the resolution of the cone photoreceptor mosaic (Westheimer, 1975). An example is vernier acuity whereby normal observers are able to identify offsets in alignment that are smaller in visual angle than the resolution limit for sinusoidal gratings. Crowding, on the other hand, occurs in the normal periphery and refers to impaired recognition or detection of a target when it is flanked by distractors. For example, a letter presented in isolation in the peripheral field is easier to identify than a letter “crowded” by adjacent letters or bars (for an overview, see Levi, 2011; **Figure 1A**). Observers with strabismic amblyopia have been found to exhibit impaired vernier acuity (Levi and Klein, 1982, 1985) and crowding in central vision (Stuart and Burian, 1962). Similar results have been reported for observers with anisometropic amblyopia; however, the deficits tend to be less pronounced (Levi and Klein, 1982, 1985; Levi et al., 1987). Spatial processing deficits of this type have been conceptualized in terms of undersampling (insufficient cortical neurons; Levi and Klein, 1986; Levi et al., 1987), spatial disarray (elevated internal noise of cortical neurons; Hess and Field, 1993; Demanins et al., 1999) and abnormal lateral interactions (Polat et al., 1997; see **Figure 2** for schematic representation of concepts). These effects are thought to occur within V1. One or more of these proposed deficits may contribute to “positional uncertainty” when viewing through an amblyopic eye. Additional mechanisms may also influence spatial processing in amblyopia. For example, saccadic eye movements and attention that have recently been proposed as a basis for crowding in normal vision (Nandy and Tjan, 2012).

**FIGURE 3:**
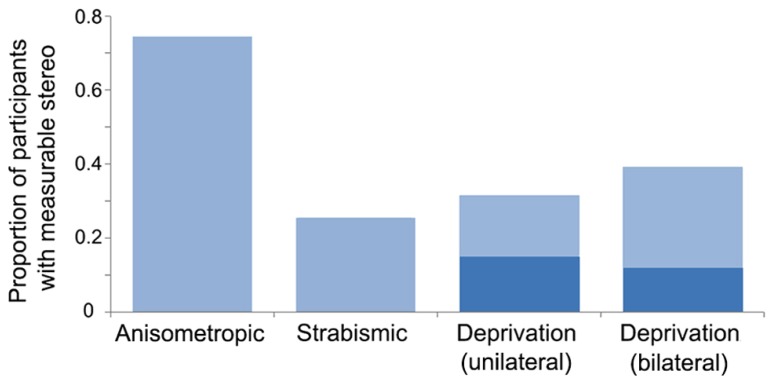
**Proportion of participants with measurable stereopsis by amblyopia type.** Data were compiled from 12 studies within which stereopsis was quantified for each participant. Measurable stereopsis was defined as 800 s of arc or better as some studies did not measure coarser thresholds. Where amblyogenic factors were mixed, designations were made based on a hierarchical categorization in the following order: deprivation, strabismic then anisometropic. The dark blue regions within the deprivation datasets represent participants who had dense, congenital cataracts. Data from the following studies were included: Anisometropic (*n* = 55; Agrawal et al., 2006; Li et al., 2011; Narasimhan et al., 2012), strabismic (*n* = 47; Agrawal et al., 2006; Hess et al., 2009; Dallala et al., 2010; Narasimhan et al., 2012; Zhou et al., 2013), deprivation (unilateral *n* = 38, of which 20 were congenital, and bilateral *n* = 68, of which 42 were congenital; Tytla et al., 1993; Hwang et al., 1999; Zubcov et al., 1999; Robbins et al., 2010, 2012; Ing, 2011; Jeon et al., 2012).

The current literature suggests that a difference in blur between the eyes during development (anisometropic amblyopia) creates less positional uncertainty than decorrelated images (strabismic amblyopia). However, the presence or absence of binocular vision may be more important than the amblyogenic factor (Levi and Klein, 1986; McKee et al., 2003). For example, McKee et al. (2003) assessed a cohort of 427 participants which included patients with strabismic, anisometropic, deprivation amblyopia, and non-amblyopic participants, and found that those without binocular function (most often those with strabismic amblyopia) exhibited greater losses in optotype and vernier acuity relative to grating acuity. A follow-up study found that crowding and the presence of stereopsis were strongly associated among a group of 72 observers with anisometropic, strabismic, and mixed amblyopia and non-amblyopic controls (Greenwood et al., 2012). Together, these studies suggest that binocular input supports the development of precise spatial signaling within V1. The effects of deprivation on hyperacuity and crowding have not been investigated to the same degree as in strabismic and anisometropic cases. However, as mentioned in section “Introduction,” measurable binocular function (stereopsis) is uncommon in both unilateral and bilateral deprivation, particularly when cases are congenital (see **Figure 3** for an overview of stereopsis by amblyopia type). This suggests that deficits related to hyperacuity and crowding may be impaired to a greater extent than grating acuity, as is the case with non-binocular anisometropic and strabismic amblyopes.

#### Second-order spatial processing

In addition to deficits in tasks requiring the local processing of first-order (luminance defined) stimuli, as have been discussed so far, abnormal processing of second-order (for example, contrast-defined) spatial stimuli has also been reported. Mansouri et al. (2005) found that observers with unilateral strabismic and anisometropic amblyopia were poorer than controls in judging the orientation of second-order stimuli, when luminance-based deficits were accounted for. Interestingly, they found that the deficit was present for both the amblyopic and the fellow eye implicating an impairment affecting the extra-striate visual cortex where cells tend to be binocular.

### LOCAL TEMPORAL PROCESSING

The effect of amblyopia on the detection and processing of local changes over time (temporal vision) appears to be less pronounced than the effect on spatial vision. This has been studied primarily using counterphasing or drifting gratings, although a range of other stimuli have been employed (see **Figure 1B** for examples of stimuli commonly used in studies of temporal vision in amblyopia).

#### First-order temporal processing

Preliminary psychophysical work suggested that the detection of counterphasing or drifting Gabors (see **Figure 1B** middle and right for schematic examples of the stimuli) was not impaired by strabismic amblyopia when deficits in spatial processing were accounted for (Hess et al., 1978b; Hess and Anderson, 1993). Subsequent studies have generally supported this finding whereby many observers with strabismic and anisometropic amblyopia have performed normally on temporal processing tasks that allow for spatial deficits to be taken into account (Hess et al., 2006; Qiu et al., 2007; Thompson et al., 2011). Deprivation cases with more severe spatial deficits, however, do appear to show some additional temporal processing abnormalities (Hess et al., 1981; Birch et al., 1993). To separate spatial from temporal deficits in deprivation amblyopia, Ellemberg et al. (1999, 2000) asked participants to detect flicker in an unpatterned luminance patch (see **Figure 1B**, left for a schematic example of the stimulus). They found small deficits at low temporal frequencies, which were similar for unilateral and bilateral cases (Ellemberg et al., 1999, 2000). Similarly, when directional tasks were employed, deficits were more pronounced at slow velocities (Ellemberg et al., 2002, 2005). Taken together, local temporal and motion processing appear to be largely intact, and certainly less impaired than local spatial processing in all types of amblyopia. When moderate deficits have been reported, they are in cases with severe local spatial deficits, and are limited to low temporal frequencies and slow velocities.

#### Second-order temporal processing

Temporal processing of second-order stimuli shows a different pattern. Simmers et al. (2011) found that perception of a variety of second-order temporal stimuli (defined by contrast, length, orientation, or polarity) was impaired in the amblyopic eye of participants with strabismic amblyopia compared to controls (first-order temporal processing was relatively unimpaired). Variability was large between the three patients tested, but task performance was not associated with deficits in spatial vision suggesting the effect was not due to visibility of the stimuli. Additionally, a deficit was also noted for the fellow eye of one participant, again suggesting the involvement of extra-striate visual areas. Comparable results have been reported for deprivation amblyopia whereby direction discrimination for second-order contrast-modulated gratings was impaired in amblyopic eyes relative to controls (Ellemberg et al., 2005), and a trend toward larger deficits in unilateral cases was observed (Ellemberg et al., 2005).

### SUMMARY

Amblyopia has a significant impact on cortical processing of local spatial information. Unilateral amblyopia results in greater losses in spatial vision in the affected eye than otherwise comparable bilateral amblyopia. This additional deficit in unilateral cases likely reflects competitive or inhibitory processes between inputs from the two eyes during development (Birch et al., 1998). Deficits in hyperacuity and crowding have also been linked to binocularity with non-binocular observers demonstrating greater impairments than would be expected based on their grating acuity. The effect of amblyopia on local motion processing is less pronounced, with only minor deficits reported for first-order stimuli in severe cases of both unilateral and bilateral deprivation amblyopia. However, perception of second-order spatial and temporal stimuli appears to be poor across all types of amblyopia, with deficits present for both amblyopic and fellow eyes. Processing of second-order motion may rely, at least in part, on anatomical loci beyond V1 (for example, Dumoulin et al., 2003). Therefore, it is possible that deficits in processing second-order stimuli involve abnormal function of extra-striate visual areas.

## GLOBAL DEFICITS IN AMBLYOPIA

### GLOBAL MOTION

#### Background

Sensitivity to motion is present throughout the visual pathway; however, the accurate representation of complex, moving objects or surfaces often requires integration across extended regions of the visual field. This is due to a phenomenon known as the aperture problem, whereby the motion direction of an edge will always be seen as perpendicular to the orientation of the edge when viewed through an aperture. Therefore, cells with small receptive fields, which sample the retinal image through small apertures, will often provide ambiguous motion direction signals. Integration across multiple small receptive fields is required to recover the true motion of the stimulus being observed (for example, Adelson and Movshon, 1982). Cells within V1 are thought to signal ambiguous “local” or “incoherent” motion due to the aperture problem, and it has been proposed that cells within dorsal extra-striate regions of the visual cortex such as area V5 (in humans) or MT (in primates) integrate these signals to reconstruct the “global” or “pattern” motion of moving objects (Allman et al., 1985; Movshon et al., 1985; Newsome and Pare, 1988; Rodman and Albright, 1989; Salzman et al., 1992; Heeger et al., 1999; Braddick et al., 2001). Areas downstream from V5/MT, such as MST, further support motion integration for particularly complex patterns of local motion such as those resulting from expansion, contraction, and rotation (Saito et al., 1986; Tanaka et al., 1986, 1989; Tanaka and Saito, 1989; Duffy and Wurtz, 1991; Britten and van Wezel, 1998).

Much of the evidence for the distinction between local and global motion comes from studies that have used either global dot motion or plaid stimuli to isolate motion integration mechanisms. Global dot motion tasks typically employ random dot kinematograms (RDKs) which are made up of two populations of moving dots; a “signal” and a “noise” population (Newsome and Pare, 1988). Signal dots move in a common direction, whereas noise dots move randomly. The observer’s task is to indicate the direction of the signal dots. The ratio of signal-to-noise dots in the stimulus is varied to measure a motion coherence threshold, which provides an estimate of the signal-to-noise ratio required for a particular level of task performance. Theoretically, cells in V1 provide information relating to the motion of individual dots, whereas cells within V5/MT are able to integrate information from V1 to resolve the global motion of the stimulus. This idea is supported by studies in primates demonstrating that MT responds to the global motion of RDKs (Newsome et al., 1989; Britten et al., 1992, 1993), that stimulation of MT can influence the perceived direction of RDKs (Salzman et al., 1992) and that MT lesions impair motion coherence thresholds (Rudolph and Pasternak, 1999). Comparable results have been found in humans (Braddick et al., 2001; Vaina et al., 2005; Thakral and Slotnick, 2011).

**FIGURE 4:**
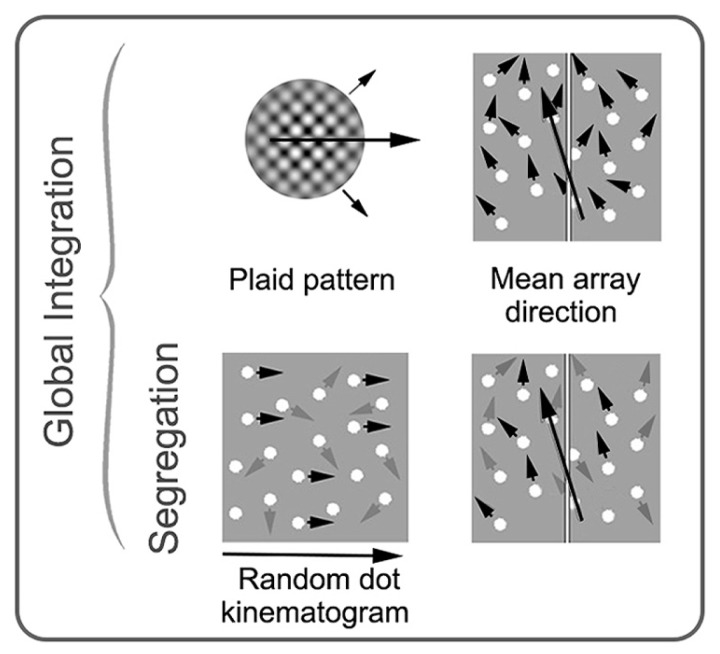
**Schematic examples of stimuli commonly used in studies of global motion perception in amblyopia.** Stimuli on the top row require integration of local motion, while the stimuli on the bottom row require both integration and segregation from noise. Small black arrows represent direction of signal elements (with prescribed motion trajectories), and gray arrows represent direction of noise elements (with random motion trajectories). The large black arrows depict the global motion direction. In a plaid pattern, two drifting gratings with different trajectories (shown with small black arrows) are combined to generate the percept of a new global motion direction. In an RDK, the proportion of signal elements to noise elements is varied. In mean array direction tasks, the mean direction of signal elements and the standard deviation around the mean is varied. These stimuli can be used with or without noise elements. RDK and mean array examples are shown here with spatially broadband dots as the elements, but dots can be substituted for narrowband elements. See text for more details. Modified from Mansouri and Hess (2006), Newsome and Pare (1988), and Thompson et al. (2008b).

Plaid stimuli are constructed from two superimposed gratings that drift in different directions (Adelson and Movshon, 1982). If the low-level properties (spatial frequency, contrast, speed, etc.) of the two “component” gratings are sufficiently similar, they will cohere and generate the percept of a single surface moving in a direction that can be distinct from either of the two component grating directions. Cells that respond to the integrated, “coherent” motion direction of plaid stimuli have been found in the pulvinar (Merabet et al., 1998), V1 (Guo et al., 2004) and a number of extra-striate areas (for example, Gegenfurtner et al., 1997); however, MT appears to have a concentration of such cells suggesting that this region has a particular specialization for motion integration (Movshon et al., 1985; Rodman and Albright, 1989; Rust et al., 2006). Brain imaging and brain stimulation studies in humans are broadly consistent with the animal neurophysiology data (Castelo-Branco et al., 2002; Huk and Heeger, 2002; Villeneuve et al., 2005, 2012; Thompson et al., 2009). See **Figure 4** for examples of global motion stimuli.

#### Unilateral amblyopia

Both RDKs and plaid stimuli have been used to investigate global motion processing in observers with amblyopia. The first study to explore global motion perception in patients with unilateral strabismic and/or anisometropic amblyopia was conducted by Simmers et al. (2003) using RDKs made up of first- or second-order (contrast-defined) dots. To assess the relative effects of contrast sensitivity and global motion deficits on motion coherence thresholds, Simmers et al. (2003) measured thresholds across a range of dot contrasts. Relative to controls, amblyopic eyes exhibited elevated motion coherent thresholds that could not be accounted for solely by a loss of contrast sensitivity. Although variable across participants, on average this effect was moderate for first-order dots (amblyopic eye thresholds ~1.5 times poorer than controls) and more pronounced for second-order dots (~4 times poorer than controls). These data provided evidence for a specific deficit in global motion perception in amblyopia. Similar effects were also found for the fellow eyes of amblyopes (thresholds elevated by ~1.4 times compared to controls). This finding further supports the presence of an extra-striate deficit in global motion processing that is independent of local processing deficits that may affect the visibility of the stimulus elements.

A number of studies have built upon this original investigation and found that the motion coherence threshold deficit in observers with amblyopia extends to radial and rotational motion (Simmers et al., 2006; Aaen-Stockdale et al., 2007). Furthermore, this deficit is not reliant on the spatial properties of the dots within the RDKs (Aaen-Stockdale and Hess, 2008) and is not dependent on the relative spatial and temporal offsets of the dots (Knox et al., 2013). A number of studies also observed deficits in fellow eyes relative to control eyes supporting Simmers et al.’s (2003) original finding (Ho et al., 2005; Aaen-Stockdale and Hess, 2008).

Complementary evidence for impaired extra-striate processing of motion signals in patients with amblyopia has also been found using briefly presented, low-contrast drifting Gabor stimuli with an abrupt onset. Impaired spatial summation of these stimuli that was independent of contrast sensitivity deficits was found for the amblyopic eyes of observers with strabismic amblyopia (Thompson et al., 2011). Furthermore, reduced activation within area MT/V5 has been reported for strabismic and anisometropic amblyopic observers using functional MRI (fMRI; Bonhomme et al., 2006; Ho and Giaschi, 2009) and impairments in tasks requiring the processing of motion signals within RDKs over large spatial offsets (maximum motion displacement or “Dmax” thresholds) have also been reported. Depending on the stimulus parameters, reduced Dmax thresholds for RDKs may reflect impaired motion processing at early stages of the visual pathway and/or abnormalities within higher level areas such as area V5/MT and downstream areas of the posterior parietal lobe that are involved in feature tracking (Ho et al., 2005; Ho and Giaschi, 2007, 2009). Somewhat unexpectedly, non-binocular amblyopes have been found to exhibit superior Dmax thresholds to binocular amblyopes (Ho and Giaschi, 2007).

On the basis of this evidence, it would appear that global motion processing is selectively impaired in amblyopia. However, the deficits seen for motion coherence thresholds are not apparent for tasks that target only the integration of local motion signals. For example, Hess et al. (2006) found normal performance for both amblyopic and fellow eyes when presented with a task that required the integration of multiple motion trajectories. Specifically, observers viewed a field of moving dots within which each dot had a slightly different motion direction and the observer had to judge the average motion direction of the dot field (a schematic is shown in **Figure 4**). The difficulty of the task was varied by manipulating the standard deviation of the individual motion directions. This task differed from the motion coherence threshold tasks as it required only integration of multiple motion signals and did not require segregation of signal from noise. Similar results have been found for plaid stimuli, whereby amblyopic and fellow eyes do not exhibit significant deficits in integrating the two component gratings into the coherent percept of a single moving surface (Thompson et al., 2008a; Tang et al., 2012).

On balance, the current data suggest that global motion perception is impaired in observers with amblyopia when measured using tasks that require signal/noise segregation but not when measured using tasks that only require integration such as plaids or variable direction dot fields (Mansouri and Hess, 2006; Thompson et al., 2008a). In other words, global motion processing (at least that requiring signal from noise segregation) is not simply an extension of local processing deficits, but appears to be impaired in its own right. This is consistent with neurophysiological data from primates with experimentally induced amblyopia (El-Shamayleh et al., 2011). Cells within MT were less tolerant of noise within RDKs when driven by the amblyopic eye but no differences were found in the responses to plaid stimuli. It should be noted that comparisons were made between amblyopic and fellow eyes in this study as this allowed for a within-subjects design to be adopted. However, as described above, both eyes can exhibit abnormal motion coherence thresholds in amblyopic observers and a similar effect has been found for strabismic (but not anisometropic) primates (Kiorpes et al., 2006). Furthermore, the recordings were made under general anesthesia, which is known to affect the response of cells to global motion stimuli (Pack et al., 2001; Guo et al., 2004). Therefore, the full extent of functional loss within MT may have been greater for these animals than the amblyopic/fellow eye comparisons suggest.

A recent, human fMRI study has provided preliminary data that may help to place the pattern of global motion deficits and abilities exhibited by observers with amblyopia within the broader context of compensatory neural networks (Thompson et al., 2012). In this study, control observers and observers with strabismic or mixed strabismic/anisometropic amblyopia viewed coherent plaids (perceived as a single moving surface) and incoherent plaids (perceived as two gratings drifting over one another) during fMRI. Importantly, the way in which the plaids were perceived did not differ among control, fellow, and amblyopic eyes. For control participants, regions throughout the extra-striate visual cortex responded differentially to coherent and incoherent plaids, in agreement with previous studies (Castelo-Branco et al., 2002; Huk and Heeger, 2002; Villeneuve et al., 2012). Responses were significantly weaker than controls when observers with amblyopia viewed the stimuli through their fellow eye, although the general pattern of activation was similar with area V5/MT exhibiting differential responses for coherent compared to incoherent plaids. However, when the stimuli were viewed through amblyopic eyes the responses were weaker still and, most importantly; V5/MT was not differentially activated. This suggests that other regions may be involved in supporting normal perception of plaids viewed with an amblyopic eye. Candidate areas identified in this study included Ventral V3 and the pulvinar. Although the sample size of this study was small (*n* = 6), the role of compensatory networks in supporting global processing in amblyopic is plausible. In the case of global motion perception, lesions of V5/MT result in elevated motion coherence thresholds but do not appear to result in lasting impairments for tasks requiring motion integration in the absence of noise (Baker et al., 1991; Rizzo et al., 1995; Rudolph and Pasternak, 1999). Presumably, the neural networks that compensate for lost V5/MT function after a lesion are able to support motion integration but not the segregation of signal from noise. A comparable situation may exist within the visual cortex of patients with strabismic and/or anisometropic amblyopia.

#### Comparison of unilateral to bilateral cases

Impaired motion coherence thresholds have also been found for observers with deprivation amblyopia caused by congenital cataracts that were present from birth (Ellemberg et al., 2002; Constantinescu et al., 2005; Hadad et al., 2012). Observers with unilateral deprivation amblyopia exhibited motion coherence threshold elevations in both eyes that are comparable to those reported by Simmers et al. (2003) for strabismic, anisometropic, and mixed amblyopes (~1.6 time poorer than normal; Ellemberg et al., 2002). However, observers who had bilateral congenital cataracts had more profound deficits in performing motion coherence tasks with thresholds in each eye being ~5 times poorer than controls (Ellemberg et al., 2002). These deficits were independent of low-level deficits such as visual acuity and contrast sensitivity, implying an extra-striate locus for the deficit (Ellemberg et al., 2002; Constantinescu et al., 2005; Aaen-Stockdale et al., 2007). Conversely, developmental cataracts which allow clear vision early in life do not appear to elevate motion coherence thresholds even when the cataracts are bilateral (Ellemberg et al., 2002). This suggests that the development of global motion mechanisms within the extra-striate visual cortex requires a period of visual input after birth and that some visual input (monocular congenital cataract) is better than none (bilateral congenital cataract).

#### Use of global motion tasks in suppression measurements

Recently, RDKs have also been used to explore the role of interocular suppression in strabismic, anisometropic, and mixed amblyopia. Mansouri et al. (2008) presented signal dots to one eye of observers with strabismic amblyopia and noise dots to the other to assess whether binocular combination was possible in these patients. The rational was that motion coherence thresholds would only be measurable under dichoptic (separate stimuli to each eye) viewing conditions if the signal and noise dots were combined within binocular areas of the visual pathway. When dots of equal contrast were presented to the two eyes, motion coherence thresholds were strongly biased toward the fellow eye whereby thresholds were very low when signal was presented to the fellow eye and noise to the amblyopic eye and very high or unmeasurable when noise was presented to the fellow eye and signal to the amblyopic eye. These results indicated suppression of the amblyopic eye. However, by reducing the contrast of the dots presented to the fellow eye, Mansouri et al. (2008) were able to demonstrate normal binocular combination in strabismic amblyopes, whereby motion coherence thresholds remained the same irrespective of which eye saw signal and which eye saw noise. In other words, binocular mechanisms were present in these observers but suppressed under normal viewing conditions. Furthermore, the contrast offset required to reach this “balance point” between the two eyes varied across observers and provided an objective measure of the extent to which the amblyopic eye was suppressed.

Since Mansouri et al.’s (2008) original study, dichoptic RDKs have been used to measure suppression in patients with strabismic, anisometropic, and mixed amblyopia (Black et al., 2011, 2012; Li et al., 2011, 2013a,b; Narasimhan et al., 2012). The results have shown that deeper suppression is related to poorer amblyopic eye acuity, poorer stereopsis, and less favorable outcomes from occlusion therapy (Li et al., 2011, 2013b; Narasimhan et al., 2012), although prospective studies are required to provide a stronger test of this effect. Furthermore, training aimed at reducing suppression of the amblyopic eye using dichoptic RDKs or modified dichoptic videogames has been found to improve both stereopsis and monocular visual acuity in adults and children with amblyopia (Hess et al., 2010a,b; Knox et al., 2011; To et al., 2011; Birch, 2013; Li et al., 2013c). As a whole, this body of work suggests that binocular interactions may play a key role in strabismic and anisometropic amblyopia (Hess et al., 2011; Hess and Thompson, 2013). This is consistent with a number of studies reporting less pronounced elevations in monocular motion coherence thresholds for amblyopic observers with residual binocular function (Ho et al., 2005; Knox et al., 2013). The question of whether suppression also plays a role in deprivation amblyopia is still open; however, very recent measurements made in our laboratory suggest that suppression is measureable in at least some cases of deprivation amblyopia using the dichoptic RDK technique.

### GLOBAL FORM

#### Background

Similar to global motion, global form perception requires the integration of local cues. However, since there is no form equivalent of the aperture problem, the distinction between V1 and extra-striate processing is less clear. Converging evidence from animal electrophysiology (Gallant et al., 1993, 1996; Pasupathy and Connor, 1999, 2001; Nandy et al., 2013) and human fMRI (Braddick et al., 2000; Wilkinson et al., 2000; Altmann et al., 2003; Conner et al., 2007) suggests that integration of local form cues involves V2 and V4, with V4 neurons signaling complex form information such as curvature and hyperbolic shapes. Stronger responses to stimuli containing forms compared to stimuli containing randomly oriented elements have been found in V1, V2 (Conner et al., 2007), VP, V4, and LOC (Altmann et al., 2003). This suggests that form processing involves a distributed network of neural areas which is likely to include feed-back and feed-forward connections between the primary and ventral extra-striate visual cortex. These concepts are under continued investigation (for example, Moratti et al., 2013).

There are a number of ways in which global processing of form has been assessed in amblyopia research. One approach involves tasks that require the binding of elements over space. Contour integration falls into this category and was a common paradigm in early amblyopia research. In these tasks, Gabor patches are oriented to produce paths or shapes that become apparent if the stimulus elements are integrated across the visual system (Field et al., 1993). Task difficulty is controlled by varying the alignment of the signal patches that form the contour (jittering the position or orientation), changing the density of the elements making up the target, or adding randomly oriented patches (noise). (Note the similarity to what we have described as local positional uncertainty, overviewed in **Figure 2**.) In a related task, individual elements (“inducers”) can be used to create illusory contours and shapes, such as those described by Kanizsa (1976). Glass patterns (Glass, 1969) can also be used to assess form perception. These stimuli are made up of pairs of dots or dipoles. A subset of the dipoles (signal) can be aligned to form a coherent pattern, while another subset of dipoles (noise) can be randomly oriented. In this task, the proportion of signal to noise is adjusted to obtain a threshold. Finally, as an alternative to tasks requiring the integration of distinct elements, observers can be asked to detect perturbations in simple forms such as circles. Such tasks typically employ radial frequency patterns that allow for precise control over the spatial frequency content of the stimulus and amount of form perturbation (Wilkinson et al., 1998). Tasks involving Glass and radial frequency patterns likely rely at least in part on ventral area V4 (Wilkinson et al., 1998, 2000; Wilson and Wilkinson, 1998). More controversy exists about the anatomical basis for contour integration and illusory contours, although they are generally thought to rely on ventral extra-striate processing (Ffytche and Zeki, 1996; Lee and Nguyen, 2001; Halgren et al., 2003; Robol et al., 2012).

**FIGURE 5:**
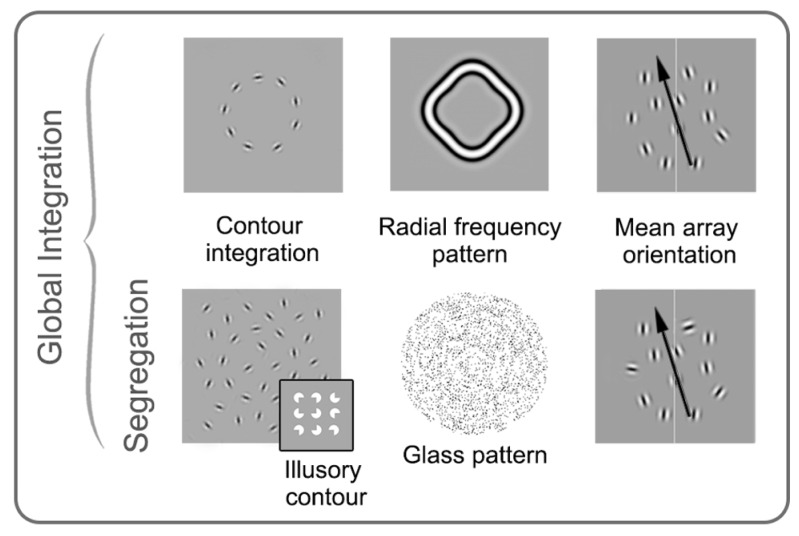
**Schematic examples of stimuli commonly used in studies of global form perception in amblyopia.** Stimuli on the top row require integration of orientation over space, while the stimuli on the bottom row require both integration and segregation from noise. Contour integration, mean array orientation, and radial frequency pattern examples are shown here made up of spatially narrowband elements, whereas the glass pattern and illusory contour examples are spatially broadband. The four examples on the left (as well as the insert) require the observer to detect or discriminate shapes or patterns, whereas in mean array orientation tasks, the mean orientation of signal elements and the standard deviation around the mean is varied, and observers are asked to judge the average orientation. See text for more details. Modified from Mansouri and Hess (2006), Levi et al. (2007), Lewis et al. (2002), Dallala et al. (2010), Mansouri et al. (2004), Putzar et al. (2007).

Although each of these tasks is thought to primarily rely on global processes, it is difficult to separate local positional uncertainty from the global form component. Mean orientation arrays allow the spatial component to be minimized, while still requiring global integration (Dakin, 2001). These tasks use a group of elements (typically multiple Gabor patches) which are oriented clockwise or anticlockwise (right or left) of the vertical midline. Observers indicate whether the overall orientation is clockwise or counter-clockwise of vertical and task difficulty is manipulated by altering the mean and standard deviation of the element orientations. More recently, there has been interest in developing stimuli which allow for direct comparisons between dorsal and ventral functions. Such stimuli typically employ orientation or direction averaging, include noise and have a temporal component to match parameters between form and motion tasks (for example, Simmers et al., 2005; Mansouri and Hess, 2006; Husk and Hess, 2013). Many other examples of global form stimuli exist, but those described above are the most commonly used in amblyopia research (see **Figure 5** for a summary).

The main challenge for measurements of global form perception in amblyopia is to separate global processing deficits from the rather extensive deficits in local spatial processing including impaired acuity, contrast sensitivity, and positional uncertainty. For example, Robol et al. (2012) have recently emphasized the importance of crowding in global tasks involving contour detection in noise. Typically, acuity and contrast sensitivity are accounted for by equating the individual elements for visibility using the fellow eye or control eyes as a reference; however, deficits relating to positional uncertainty are more difficult to control for.

#### Unilateral amblyopia

One of the first studies to investigate spatial integration in amblyopia measured the effect of flanking stimuli on the detection of a centrally presented Gabor patch (Polat et al., 1997). Collinear flankers facilitated task performance for controls; however, this effect was absent or even reversed in observers with strabismic and/or anisometropic amblyopia. This led to the suggestion that global contour integration mechanisms may be abnormal in amblyopia. Subsequent studies have supported this idea. For example, experiments investigating contour detection in noise found evidence for specific deficits in global integration relative to controls in strabismic (Kovács et al., 2000; Mussap and Levi, 2000) and anisometropic amblyopes (Chandna et al., 2001) as well as non-amblyopic participants with ocular misalignment (Kovács et al., 2000). However, this is not a ubiquitous finding. Hess et al. (1997) found that the deficits exhibited by strabismic amblyopes on a contour detection task could be accounted for by impairments in judging the local position of the stimulus elements rather than global integration. Furthermore, this group reported almost no contour integration deficits in observers with anisometropic amblyopia (Hess and Demanins, 1998).

Building on these earlier studies, Levi et al. (2007) assessed the ability of observers with strabismic and anisometropic amblyopia to discriminate between a circle and an ellipse made up of oriented Gabor patterns. Position and radius of the shapes were kept constant to minimize the requirement for positional accuracy. A mild deficit (~1.4 times worse than controls) remained when contrast sensitivity was carefully accounted for indicating the presence of a measureable global form processing deficit for this specific task. This effect was only present in the amblyopic eye, was more pronounced for strabismic than anisometropic viewers (which was related to the presence of binocularity), and was particularly evident when contours were presented in noise. Deficits in global form perception relating to deficits in extracting signal from noise have also been reported by studies employing Glass patterns. In particular, abnormal perception of Glass patterns has been reported for amblyopic eyes in both deprivation (Lewis et al., 2002) and strabismic amblyopia (Rislove et al., 2010). Together, it seems that normal binocular function may play a role in the development of mechanisms involved in signal/noise segregation for form.

However, signal/noise segregation is not sufficient to account for all global form deficits reported for observers with amblyopia. For example, deficits in the perception of radial frequency patterns have been reported in the amblyopic eyes of small groups of adults (Hess et al., 1999; Dallala et al., 2010), and a large group of children (Subramanian et al., 2012) with strabismic amblyopia. A similar result was found for participants with deprivation amblyopia (Jeffrey et al., 2004). This supports the idea that the processing of contours and shapes is influenced by amblyopia even in the absence of noise, but does not rule out positional uncertainty as the cause of the processing deficit (Dallala et al., 2010). Positional uncertainty was accounted for by Popple and Levi (2000) who showed that a variety of global alignment illusions were not perceived in observers with strabismic and anisometropic amblyopia indicating the presence of a global form deficit over and above local processing abnormalities.

Performance in mean orientation tasks without noise elements have also been investigated for observers with amblyopia. Simmers and Bex (2004) found that both the amblyopic and the fellow eyes of observers with strabismic or anisometropic amblyopia were impaired on a mean array orientation task relative to controls. This effect could not be replicated in controls even if the number, visibility (contrast), and orientation variance of the elements were matched to the low-level deficits shown by amblyopic observers. However, Mansouri et al. (2004) used a similar task and found no integrative deficits. They calibrated the contrast of the Gabor patches displayed to each eye to equate stimulus visibility and found no additional global form deficit in either eye of observers with strabismic or anisometropic amblyopia. This was also the case for a global form task involving second-order elements (Mansouri et al., 2005). Deficits for first-order orientation integration were later found by this group only when randomly oriented noise elements were added to the array (Mansouri and Hess, 2006) which resonates with the pattern of deficits found for global motion processing, and the idea of a generic signal/noise segregation deficit. Recently, a comprehensive investigation of global orientation coherence was conducted whereby observers judged the average orientation of fields of elements created by filtering white noise to generate spatially narrowband oriented Gabor-like patterns, with each element having a limited lifetime (Husk and Hess, 2013). The stimuli contained both signal and noise elements. By varying both the signal-to-noise ratio within the stimuli and the orientation bandwidth of the elements, Husk and Hess (2013) revealed subtle deficits in both eyes of strabismic and anisometropic amblyopes that were primarily due to deficits in judging local orientation rather than abnormal global processing. In general, therefore, it would appear that any specific global form processing deficit in unilateral amblyopia is quite mild when positional uncertainty and signal/noise segregation are taken into account.

#### Comparison of unilateral to bilateral cases

There are fewer studies that have evaluated global form perception in cases of bilateral amblyopia. Lewis et al. (2002) found that observers with a history of bilateral deprivation were poorer at detecting global form in Glass patterns than controls (by a factor of ~1.7), and those with unilateral deprivation amblyopia (unilateral cases were ~1.3 times worse than controls on the same task). However, differences between observers with unilateral and bilateral deprivation amblyopia were not apparent for a task involving radial frequency patterns (Jeffrey et al., 2004). Another group used an illusory contour task and found that although the shapes “popped out” of the stimulus arrays for control observers (indicating global from processing), patients with a history of early bilateral deprivation appeared to process the stimuli in serial, searching for four elements with inwardly facing cut outs (Putzar et al., 2007). Putzar et al. (2007) did not include unilateral cases. Overall, it would appear that bilateral deprivation does result in global form processing deficits that may be more pronounced that those caused by unilateral deprivation for particular form-based tasks, such as those containing noise. However, as emphasized by Lewis et al. (2002), it is notable that observers with bilateral amblyopia exhibit more pronounced deficits for global motion (~5-fold elevations in threshold; Ellemberg et al., 2002) than for global form (~1.7-fold elevations in threshold; Lewis et al., 2002).

#### Use of global form tasks in suppression measurements

As with global motion, global form tasks have also been used to measure suppression and binocular combination in cases of unilateral strabismic and/or anisometropic amblyopia with signal elements presented to one eye and noise to the other (Mansouri et al., 2008). Varying interocular contrast in favor of the amblyopic eye allows for form information to be combined between the two eyes as it does for RDKs, indicating that suppression acts upon both form and motion processing. A recent study directly compared “balance point” contrasts for form and motion tasks and found that suppression was more pronounced for motion than form processing (Zhou et al., 2013). This may reflect a greater susceptibility of global motion processing to suppression; however, a prospective comparison between monocular form and motion coherence thresholds and the strength of suppression is required to address this question.

### SUMMARY OF GLOBAL MOTION AND FORM

Despite the use of varied paradigms, several patterns emerge from studies investigating global form and motion perception in amblyopia. For global motion, there is compelling evidence for a specific deficit in addition to those inherited from abnormal local processing. For global form, the evidence is less convincing (for example, Simmers et al., 2003, 2005; Husk and Hess, 2013). This is particularly interesting in light of the evidence described above which suggests that local temporal processing is less affected by amblyopia than local spatial processing. For both global form and motion perception, when deficits are measured which cannot be accounted for by local spatial deficits, they appear to be most pronounced for tasks that require the segregation of signal from noise, suggesting that this process may be particularly susceptible to abnormal visual experience early in life (Mansouri and Hess, 2006; although see Levi, 2007). In addition, a number of studies have reported global processing deficits for both eyes of observers with unilateral amblyopia, strongly implicating abnormalities affecting binocular regions of the striate and extra-striate visual cortex. This effect is reported more often for global motion than global form tasks. Although less widely studied, patients with bilateral deprivation amblyopia appear to perform more poorly than patients with unilateral deprivation amblyopia for global tasks. This is the opposite of what one would expect based on the pattern of local processing deficits which are more severe in unilateral deprivation (Ellemberg et al., 2002; Lewis et al., 2002) and suggests that monocular input to extra-striate areas during early infancy allows for more normal development of global processing than no input at all.

### FACE PERCEPTION

#### Background

Face perception is an interesting example of the interplay between local and global cues in visual processing. Global processing of faces is thought to take precedence over local processing of individual features when observers are required to make refined within-category distinctions, such as recognizing individuals or expressions. These global processes can be disrupted by inverting faces which results in significant impairments in face processing and a reliance on local facial features (reviewed by Maurer et al., 2002).

Face detection has been associated with the right inferior temporal cortex (Haxby et al., 1991; Wilkinson et al., 2000), particularly the fusiform area (sometimes called the fusiform face area or FFA). Additionally, the N170 component of the visual evoked potential is thought to be face selective (Bentin et al., 1996). Mooney faces (Mooney, 1957) are a set of stimuli commonly used for measuring face detection, and consistently activate FFA and elicit an N170 response in controls. These stimuli are high-contrast, black and white, spatially obscured images which represent faces with varying levels of clarity.

Tasks designed to investigate discrimination of clearly visible faces typically fall into two broad categories; those targeting “invariant” and those targeting “variant” aspects of face processing (Bruce and Young, 1986; Haxby et al., 2000). Invariant cues are those that remain constant, such as the spacing between the eyes and the shape of a face. One way of measuring perception of the invariant features of faces is to test sensitivity to the relative position of features (for example, eye spacing, distance between eyes and nose). The “Jane Task” (Mondloch et al., 2002) requires observers to judge either the relative spacing of features or the shape of individual features in a single face. Observes with normal vision are more sensitive to the relative spacing of features when they are presented upright compared to inverted, whereas sensitivity to feature shape is similar regardless of orientation (Freire et al., 2000).

**FIGURE 6:**
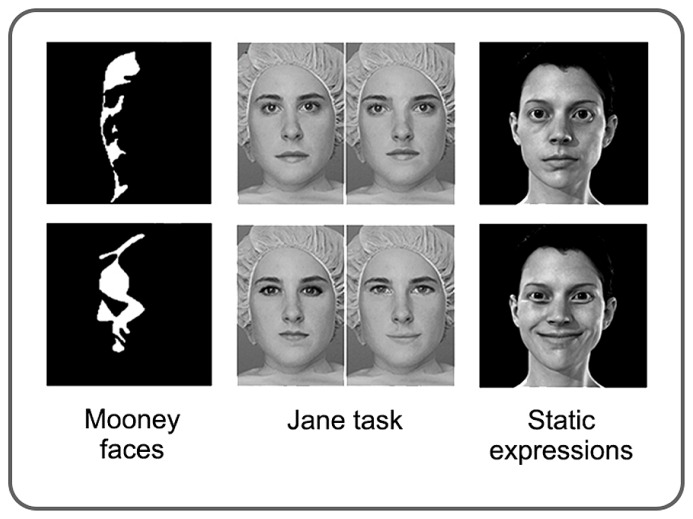
**Examples of stimuli commonly used in studies of face perception in amblyopia.** Mooney faces are high-contrast, black and white images which represent faces with varying levels of clarity. The Jane task allows for the global processing of feature spacing (top row) and the local processing of feature shape (bottom row) to be tested independently. Perception of facial expressions and speech postures is typically assessed using static images; however, the stimuli vary considerably between studies. See text for more details. Modified from Mooney (1957), Maurer et al. (2002).

Variant aspects of faces change within an individual, and include the generation of speech sounds and emotional displays. Tests of variant face processing typically involve faces with varying patterns of facial-muscle activation (see **Figure 6**). These can be dynamic, but are typically static images of transient states, such as someone smiling, or mouthing the letter “o”. Variant and invariant components of face perception may involve distinct regions of the extra-striate visual cortex with invariant processing relying more on FFA, and variant processing on the posterior region of the superior temporal sulcus (pSTS; Bruce and Young, 1986; Haxby et al., 2000). This anatomical distinction has recently been supported by a unique case of a patient who sustained a lesion that included her right pSTS. She was subsequently unable to match facial expressions, but had a generally preserved ability to identify faces (Fox et al., 2011).

#### Unilateral amblyopia

Interest in the effect of amblyopia on the development face perception began in the early 2000s with compelling papers published in *Neuron* (Lerner et al., 2003) and *Nature* (Le Grand et al., 2001). Lerner et al. (2003) presented the striking fMRI finding that although V1 activation remained relatively unchanged, fusiform activation was severely attenuated when observers with unilateral strabismic and anisometropic amblyopia viewed faces with their amblyopic eye. This deficit was not measurable for non-face categories (houses), and could not be replicated in control subjects by degrading images to simulate the poorer acuity and contrast sensitivity of amblyopic eyes (Lerner et al., 2003). Furthermore, the fMRI result was supported by psychophysical data showing significant deficits in the identification of famous faces, and, more dramatically, facial expressions for the amblyopic compared to the fellow eye (Lerner et al., 2003). Similar results have recently been reported using electroencephalography (Bankó et al., 2013a,b). However, a follow-up study by Lerner et al. (2006) found that the functional deficit in extra-striate visual areas revealed by fMRI was not face-specific and could be explained by weaker activation of the visual cortex when object identification relied on foveal vision in the amblyopic eye. Therefore, the face processing anomalies reported in their earlier paper are most easily explained by deficits in the processing of local image features.

The link between deficits in local processing and impaired face perception in unilateral strabismic amblyopia has recently been investigated using psychophysics. Cattaneo et al. (2013) found no deficits in the detection of Mooney faces in observers with strabismic amblyopia but did find poorer performance on the “Jane” task (which targets configural processing of facial features) for amblyopic eyes. However, the deficit for this task was present for both upright and inverted images. This is consistent with abnormal processing of individual local features rather than a specific impairment of configural face processing, which would not be predicted to affect the processing of inverted faces.

In contrast to unilateral strabismic and anisometropic amblyopia, strong evidence for abnormal face processing has been reported in cases of deprivation amblyopia. In unilateral cases, deprivation affecting the left eye, but not the right eye, leads to deficits in configural processing of faces that are specific to the upright version of the “Jane” task (Le Grand et al., 2003). The explanation provided for this rather unexpected phenomenon is twofold. First, the right hemisphere is specialized for face processing, and second, visual fields are restricted in infancy and displaced temporally, creating a short period during which the left eye conveys information to the right hemisphere only. Together, Le Grand et al. (2003) suggest that visual input to the right hemisphere in early infancy is essential for the normal development of configural face processing. In summary, unilateral amblyopia does not appear to affect face processing beyond what is expected from inherited local processing abnormalities, except in the very specific case of left eye congenital cataract which is thought to interfere with development of the right inferior temporal cortex.

#### Comparison of unilateral to bilateral cases

As would be expected from the deficits in the Jane task in left eye unilateral cataract described above, bilateral deprivation from birth also results in attenuation of the upright advantage in configural processing (Le Grand et al., 2001; Mondloch et al., 2010). This appears to be specific to human faces, as the same cohort was as sensitive as controls to feature spacing in houses and monkey faces (Robbins et al., 2010). Recent work has supported the presence of configural face processing impairments in observers with a history of bilateral deprivation and identified additional impairments in face recognition and recall (De Heering and Maurer, 2014). However, the detection of Mooney faces appears to remain intact (Mondloch et al., 2013) as has been reported for strabismic amblyopia (Cattaneo et al., 2013). This may be because face detection is preserved regardless of early visual experience, although additional investigation is required.

An early study bridged the gap between invariant and variant aspects of faces. Geldart et al. (2002) investigated a group of 17 patients with a history of bilateral deprivation and found that identification of faces was impaired when the stimuli were presented with different head positions or expressions. However, matching of expressions and the ability to lip read were unimpaired (Geldart et al., 2002). A subsequent study corroborated the deficit in varying head position and lighting conditions and revealed a deficit for lip reading using a more complex task (Putzar et al., 2010b). Specifically, Putzar et al. (2010b) investigated lip reading with the McGurk effect, which relies on the integration of visual and auditory speech cues. A follow-up study using fMRI found that observers with amblyopia exhibited a different pattern of cortical activation when lip reading. In particular, lip reading was associated with strong activation in the superior temporal sulcus (STS) for controls but not for patients (Putzar et al., 2010a).

As a whole, current evidence supports the presence of a specific configural, or global, face processing deficit in cases of bilateral amblyopia that may also be accompanied by abnormal face recall, and perhaps abnormal processing of the variable aspects of faces. However, evidence for face-specific deficits in unilateral strabismic and anisometropic amblyopia is equivocal and is difficult to separate from local processing impairments. This is similar to the trend described for global form processing in the previous section and, together, these results suggest that unilateral strabismic and anisometropic amblyopia do not specifically impair the processing of global spatial form.

### BIOLOGICAL MOTION

#### Background

Biological motion perception refers to the ability to extract information relating to gait, gender, and even emotional state from the movements of other people. The stimuli most commonly used in studies of biological motion perception are point light walkers (Johansson, 1973), whereby moving dots are used to represent the motion patterns of the major joints of the body. Point light walkers can be embedded in displays containing noise dots to allow for task difficulty to be manipulated (Grossman and Blake, 1999). As with variant aspects of face processing, biological motion tasks typically exhibit an inversion effect (Troje and Westhoff, 2006) suggesting that configural processing is involved, and have been found to activate pSTS (Grosbras et al., 2012). The precise mechanisms underlying biological motion perception are still being investigated. However, biological motion is likely to rely on a combination of motion integration, structure from motion (the grouping together of elements based on common or related motion trajectories) and, when noise dots are presented, signal/noise segregation. For example, biological motion perception can remain intact in patients with brain lesions that significantly impair global motion perception (Vaina et al., 1990; Jokisch et al., 2005), suggesting that biological motion relies on multiple sources of visual information, possibly from both dorsal and ventral areas of the extra-striate visual cortex.

#### Unilateral amblyopia

Three studies have investigated biological motion in anisometropic, strabismic, or mixed amblyopia. Both Neri et al. (2007) and Thompson et al. (2008b) asked observers with strabismic and/or anisometropic amblyopia to discriminate point light displays in noise. Both studies found that biological motion perception was intact in observers with amblyopia as evidenced by the presence of normal inversion effects for point light stimuli. Although amblyopic eyes did exhibit elevated thresholds relative to fellow eyes and controls, this could be attributed to a greater sensitivity to the presence of noise dots rather than a selective impairment in biological motion processing (Thompson et al., 2008b). Recently, Luu and Levi (2013) used a different approach to assess biological motion perception in observers with strabismic and anisometropic amblyopia. They presented point light stimuli representing two dancers and the observers had to decide whether the dancers were moving in or out of synchrony with one another. Task difficulty was controlled by removing dots from the point light displays, not by adding noise. Both controls and amblyopes were more sensitive to synchronous displays indicating that biological motion processing was relatively robust to amblyopia. However, both the amblyopic and fellow eyes of observers with amblyopia required more dots than controls to perform the task, a deficit that may have been due to undersampling (Levi and Klein, 1986) of the stimuli by observers with amblyopia. Therefore, although only three studies have been conducted to date, it would appear the biological motion perception is preserved in amblyopia and that poorer performance by amblyopic eyes for biological motion tasks can be attributed to signal/noise segregation or undersampling.

#### Comparison of unilateral to bilateral cases

Biological motion perception also appears to be preserved in cases of bilateral congenital deprivation, despite a substantial global motion deficit in the same observers (Hadad et al., 2012). Surprisingly, Hadad et al. (2012) found no differences between observers with deprivation amblyopia and controls even when stimuli were presented in noise.

One possible explanation for the preservation of biological motion perception is that neural systems supporting structure from motion, which are thought to combine information from both dorsal and ventral processing streams, are not affected by amblyopia. However, this does not appear to be the case. Performance on structure from motion tasks requiring the detection of non-biological objects is impaired in both the amblyopic and fellow eyes of observers with strabismic and anisometropic amblyopia (Wang et al., 2007; Hayward et al., 2011; Husk et al., 2012). Furthermore, structure from motion deficits appears to be independent from visual acuity losses (Giaschi et al., 1992) and cannot be accounted for by impaired signal/noise segregation (Husk et al., 2012). It has been proposed that structure from motion tasks involve second-order motion and form processing pathways which may explain the deficits exhibited by observers with amblyopia (Hayward et al., 2011). Another potential explanation is that the integration of form and motion is abnormal in amblyopia (Husk et al., 2012). Either way, it would appear that the preservation of biological motion perception is related to the biological nature of the task which may recruit specialized and robust neural pathways (Troje and Westhoff, 2006).

### THE EFFECTS OF BILATERAL LONG-TERM VISUAL DEPRIVATION ON GLOBAL PROCESSING

Up until this point in the review, where deprivation amblyopia has been discussed we have focussed on studies including observers who had dense cataracts that were present within the first six months of life, and treated during infancy. These studies are often able to precisely quantify the onset, severity, and duration of deprivation each of which can significantly alter the pattern of impairment in global processing (for review, see Lewis and Maurer, 2005, 2009; Maurer et al., 2007). However, there is a related literature concerned with dramatic cases of patients who regain sight after many years of bilateral deprivation. A detailed treatment of this literature is beyond the scope of this review, but a brief summary of the effects of long-term visual deprivation on global processing is of interest as a comparison to the cases described so far.

We have identified 8 peer reviewed case studies of extended bilateral deprivation that included measurements of global processing. The cases are quite varied and in some cases histories are uncertain. Patients HD (Ackroyd et al., 1974), SC (Carlson et al., 1986), and MM (Fine et al., 2003; Gregory, 2003) had at least three years of visual experience followed by at least 20 years of deprivation. Patient SB had vision for approximately 10 months followed by 52 years of deprivation (Gregory and Wallace, 1974), and PB, JA, SK, and SRD experienced deprivation from birth lasting between 7 and 29 years (Ostrovsky et al., 2006, 2009). The patients were assessed soon after the cause of the deprivation was removed with the exception of SRD who had 20 years of normal visual experience before being formally assessed (Ostrovsky et al., 2006). Across these cases, patients who had very little early visual experience tended to have more severe impairments in global tasks targeting the dorsal stream than patients who had longer periods of visual experience prior to the onset of deprivation. A different trend is apparent for global form tasks targeting the ventral stream whereby extended bilateral deprivation tends to result in persistent and functionally severe deficits in object and face recognition irrespective of the age of onset. This is highlighted in an fMRI study of patient MM who had normal activity within V5 (Gregory, 2003) in response to global dot motion but no activity within the inferior temporal cortex in response to objects (Fine et al., 2003). On the basis of this small number of cases, the development of global motion processing seems to require only a short period of visual experience after birth, whereas normal global form processing requires ongoing visual experience.

## DISCUSSION

**FIGURE 7:**
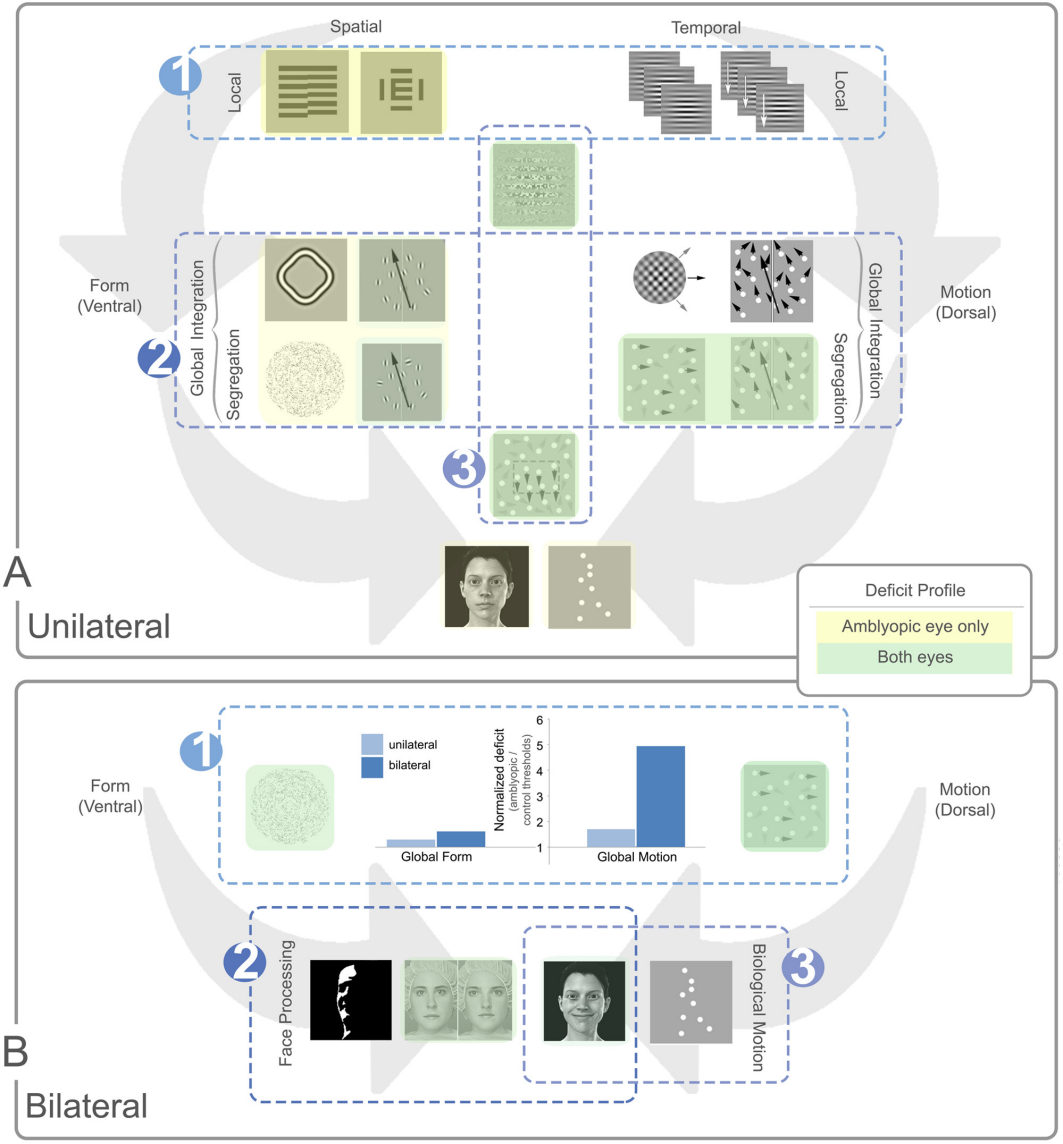
**Summary of local and global processing deficits in unilateral and bilateral amblyopia.** Panel **(A)** depicts general trends relating to unilateral amblyopia and panel **(B)** shows additional trends for bilateral amblyopia. In each panel, example stimuli are shown with spatial (ventral) tasks presented to the left of each panel and temporal (dorsal) tasks to the right. In panel **(A)** local tasks are shown at the top of the panel, global tasks in the middle, and more complex tasks at the bottom. In panel **(B)**, all tasks are global with more complex global tasks shown in the bottom row. Tasks with no highlighting are not specifically affected by amblyopia. Yellow highlighting indicates a deficit for the amblyopic eye only and green indicates a deficit for both eyes. The luminance of the color (dark or light) indicates how consistent the specific deficit is across studies, with darker colors representing consistent deficits. Three main trends are notable in panel **(A)**. **(A1)** Local spatial deficits are more pronounced than local temporal deficits. These deficits are present in the amblyopic, but generally not the fellow eye. **(A2)** Global motion tasks requiring the segregation of signal from noise show more consistent deficits in both the amblyopic and fellow eye when compared to global form tasks. These deficits do not appear to be inherited from abnormalities in the processing of local temporal information [cf. trend **(A1)**] and the deficit does not extend to tasks requiring only motion integration. **(A3)** Tasks which rely on second-order processing are impaired, an effect seen in both the amblyopic and fellow eyes. Three additional trends are apparent for bilateral cases represented in panel **(B)**. **(B1)** The dorsal stream deficit is exaggerated in bilateral cases. The bar graph shows normalized data from two separate studies comparing unilateral and bilateral amblyopia using Glass patterns for form, and RDKs for motion (Ellemberg et al., 2002; Lewis et al., 2002). Larger values on the *Y*-axis indicate a greater deficit for amblyopic eyes relative to controls. While the global form deficits are similar between unilateral and bilateral cases, the global motion deficits are much more pronounced for bilateral cases. **(B2)** Some aspects of face processing are impaired in bilateral amblyopia, for example, configural processing of identity. **(B3)** Biological motion perception, and possibly some aspects of variable face processing may be preserved after bilateral visual deprivation. See text for more details.

The literature on global processing in amblyopia includes a range of amblyopia sub-types and psychophysical tasks; however, a number of trends are apparent across studies. These are summarized in **Figure 7**.

### TRENDS FOR UNILATERAL AMBLYOPIA

The first trend relates to the differential effects of unilateral amblyopia on the local processing of spatial and temporal information. Amblyopic eyes have impaired spatial acuity and contrast sensitivity combined with crowding and impaired hyperacuity. These effects have been linked to the concept of positional uncertainty, which may reflect undersampling of the retinal image or disarray of retinotopic representations of space within the visual cortex. Other factors such as fixation instability may also contribute to these deficits in spatial vision. Conversely, the processing of local temporal information appears to be relatively unaffected by amblyopia (see **Figure 7A**-1). There is evidence that strabismic and anisometropic amblyopia affect parvocellular inputs to the visual cortex from the lateral geniculate nucleus more than magnocellular inputs (Shan et al., 2000; Mizoguchi et al., 2005; Davis et al., 2006; Hess et al., 2010c). In very general terms, the parvocellular pathway (which feeds into the ventral processing stream) is thought to primarily support spatial vision whereas the magnocellular pathway (which feeds into the dorsal processing stream) primarily supports temporal vision. Therefore, a differential effect of amblyopia on these two pathways would be consistent with the pattern of local processing deficits reported by the majority of studies in the amblyopia literature.

Somewhat unexpectedly, the pattern of local processing deficits in unilateral amblyopia is not reflected by global processing tasks targeting extra-striate visual areas. Specifically, the deficit for global motion perception is more pronounced than that for global form (see **Figure 7A**-2). Deficits are present in global form tasks, but they are small and inconsistent, possibly due to the already extensive impairments present for the local processing of form. Only a few studies have found a form processing deficit in both fellow and amblyopic eyes. In these cases, the deficit is subtle and typically only present in tasks that require segregation of signal from noise. In comparison, the impairments reported for global motion perception are more reliable across studies, and present in both the amblyopic and fellow eyes implicating an extra-striate deficit. This is particularly compelling given that local processing and global integration are both largely preserved for motion. This is generally consistent with the idea of dorsal stream vulnerability, which proposes that dorsal areas of the extra-striate visual cortex are more susceptible to the effects of abnormal development than ventral areas (Braddick et al., 2003). The neural basis of this effect is not well understood and there is considerable cross-talk between the putative dorsal and ventral streams (de Haan and Cowey, 2011; Gilaie-Dotan et al., 2013), but global motion impairments have been observed in a variety of neurodevelopmental conditions (for example, Taylor et al., 2009). Dorsal stream vulnerability is typically linked, at least theoretically, with compromised function of the magnocellular pathway. This is not consistent with the largely intact local temporal processing in amblyopia. This conundrum is yet to be resolved, although recent studies suggest that suppression of amblyopic eye inputs is stronger within the dorsal than the ventral stream (Zhou et al., 2013) which may play a role. What is clear is that amblyopia differentially affects the global processing of form and motion, demonstrating that these two visual domains respond differently to abnormal sensory input during development. From a functional perspective, abnormal function of the dorsal processing stream may influence the development of visuomotor coordination resulting in inaccurate reaching and grasping in patients with amblyopia (Niechwiej-Szwedo et al., 2011; Suttle et al., 2011).

Interestingly, local second-order spatial or temporal tasks have been found to be impaired in both amblyopic and fellow eyes. A similar trend has been observed for form-from-motion tasks which may also rely on second-order mechanisms (see **Figure 7A**-3). Although the neural systems that allow for second-order processing are not well understood, it has been suggested that extra-striate areas are involved (for example, Dumoulin et al., 2003). It is possible that second-order processing deficits in amblyopia reflect a specific extra-striate vulnerability.

### COMPARISON OF TRENDS BETWEEN UNILATERAL AND BILATERAL CASES

The comparison of unilateral to bilateral amblyopia provides further insights into the processes mediating visual cortex development. Local spatial deficits, which are extensive in unilateral amblyopia, are generally less pronounced in bilateral cases with similar onset and duration. It appears that deprivation itself impairs spatial vision, but that competition between the eyes in unilateral cases results in additional deficits for the amblyopic eye (Birch et al., 1998). A strong case has been made that suppression of the weaker by the stronger eye plays an important role in the local spatial losses associated with unilateral strabismic and anisometropic amblyopia (Mansouri et al., 2008; Li et al., 2011; Wong, 2011). However, impaired binocular interactions appear to have some role to play in bilateral cases as well. Stereopsis is generally poor in bilateral cases (see **Figure 3**), and preliminary data suggest suppression is also a factor, albeit to a lesser extent than in unilateral cases. The role of binocular function in bilateral cases is an interesting area for further investigation.

Global processing in bilateral cases shows a similar trend to unilateral amblyopia in that the dorsal stream shows more pronounced deficits. However, this effect appears to be exaggerated. Specifically, bilateral cases show motion perception deficits that are approximately four times greater than those found in unilateral cases, whereas global form deficits are similar in magnitude between unilateral and bilateral cases (see **Figure 7B**-1). Additionally, there is converging evidence that bilateral deprivation affects certain aspects of face processing, particularly those of a global nature (for example, the configural aspect of the Jane task – see **Figure 7B**-2). Together, this suggests that bilateral deprivation results in more pronounced global deficits than unilateral deprivation, particularly for global motion tasks. Interestingly, the dorsal/ventral trend is switched for amblyopia caused by extended bilateral deprivation after early visual experience, with ventral stream deficits predominating. Perhaps, early experience is both necessary and sufficient to preserve the neural architecture for global motion perception (Dormal et al., 2012), although more in-depth psychophysical evaluation of extended cases is necessary for an accurate comparison.

### PRESERVATION OF GLOBAL FUNCTIONS DESPITE ABNORMAL VISUAL DEVELOPMENT

Global functions which are preserved despite abnormal visual experience are equally valuable in helping to understand visual circuitry. In addition to local motion processing and motion intregration, biological motion and face detection are notable examples of preserved function. Although it is not surprising that biological motion perception *per se* is generally intact in unilateral amblyopia (this is broadly consistent with form and face perception), it is compelling that these perceptual skills appear to be preserved in cases of bilateral deprivation. This suggests that certain aspects of complex processing may be resilient to abnormal experience. In the case of biological motion, perhaps this resilience is due to the use of multiple sources of visual information from both the dorsal and ventral streams, or perhaps because this visual ability fulfills a particularly necessary function (Troje and Westhoff, 2006). Whether or not variant aspects of facial processing are spared in a similar way to biological motion and face detection is still an open question, and it requires more investigation (see **Figure 7B**-3).

### CONSIDERATIONS FOR FUTURE RESEARCH

Processing of the variant aspects of faces, such as emotional expressions, is a good example of how the development and refinement of psychophysical tasks would aid in the further elucidation of extra-striate deficits in amblyopia. Along with new task development, establishing standard protocols for global tasks would allow for more direct comparisons between groups. A few protocols described in this review are beginning to be standardized for possible clinical use. Examples are contour integration (Kovács et al., 2000; Chandna et al., 2001), radial frequency deformations (Subramanian et al., 2012), interocular suppression (Black et al., 2011), and the Jane task (Mondloch et al., 2002). Further progress in this area will help to improve our understanding of extra-striate function in amblyopia.

Beyond variability in task parameters, another consideration when interpreting studies in this area is the heterogeneity of the patient population being investigated. Important sources of variability have been omitted from this review to allow for a focus on the overarching themes. For example, the age at which the amblyogenic factor started to affect the child’s vision, the combination of amblyogenic factors involved, the age at which it was resolved, and the degree to which vision was degraded in the interim are all important factors. Similarly, whether or not treatment for the resulting amblyopia was administered or successful is an important source of heterogeneity. This variation is likely to impact both local and global processing, particularly for deprivation cases caused by cataract. In these cases, vision degradation is very severe if the cataracts are dense and present at birth, or quite mild if the cataracts are developmental and diffuse, with a spectrum between the two. For this reason, psychophysical studies of deprivation amblyopia are typically selective for either congenital onset and early treatment, or long-duration deprivation, and this division is reflected this review. Finally, many studies of anisometropic and strabismic amblyopia have small samples due to the extensive psychophysical testing involved.

McKee et al. (2003) conducted one of the few studies in the field with a large sample size and found that the presence or absence of binocularity was a key factor in the pattern of visual deficits experienced by patients. Recently, treatments for amblyopia focusing on improving binocular interaction have gained momentum with improvements in amblyopic eye acuity and stereopsis being reported even for adult patients (Li et al., 2013c). This approach to treatment involves the use of video games in which different game components are shown to either the weaker or stronger eye. Elements presented to the fellow eye are reduced in contrast to overcome suppression and allow for information to be combined between the two eyes as has been described in the Section “Global Motion.” A randomized, placebo controlled, clinical trial is currently underway to evaluate the efficacy of this intervention.

A key question in light of this review is the impact of amblyopia treatment on extra-striate function. For example, do acuity gains translate into improved motion perception? Very little data are currently available to address this question in unilateral amblyopia; however; initial unpublished findings suggest that occlusion therapy in children with amblyopia can result in improved in global motion perception (Anstice et al., 2013). One study of interest for the potential to improve extra-striate function was undertaken in bilateral cases. Jeon et al. (2012) found improvements in a number of global functions after 40 h of video game play. This is promising; particularly as the availability of contrast-balanced dichoptic games which may have even greater effects is increasing. The impact of amblyopia treatment on extra-striate function is particularly relevant for early and extended bilateral cases, for which the deficits are most pronounced.

Pooling what is known about the local and global deficits in the various types of amblyopia provides valuable insights into how the visual system is organized, and how this organization changes based on experience. It is becoming clear that the neurodevelopmental changes associated with amblyopia have effects that influence processing throughout the visual cortex and highlight patterns of vulnerability and resilience within the developing brain.

## Conflict of Interest Statement

The authors declare that the research was conducted in the absence of any commercial or financial relationships that could be construed as a potential conflict of interest. Benjamin Thompson is a named inventor on two patents covering a new treatment approach to amblyopia that is described in the manuscript: patent numbers US12528934 and US8006372 B2.
